# Ethnic variability associating gut and oral microbiome with obesity in children

**DOI:** 10.1080/19490976.2021.1882926

**Published:** 2021-02-17

**Authors:** Baskar Balakrishnan, Vaithinathan Selvaraju, Jun Chen, Priscilla Ayine, Lu Yang, Jeganathan Ramesh Babu, Thangiah Geetha, Veena Taneja

**Affiliations:** a Department of Immunology, Mayo Clinic, Rochester, MN, USA; b Department of Nutrition, Dietetics, and Hospitality Management, Auburn University, Auburn, AL, USA; c Division of Biomedical Statistics and Informatics, Mayo Clinic, Rochester, MN, USA; d Boshell Metabolic Diseases and Diabetes Program, Auburn University, Auburn, AL, USA

**Keywords:** Microbiome, minorities, obesity, disparity, socioeconomic factors

## Abstract

Obesity is a growing worldwide problem that generally starts in the early years of life and affects minorities more often than Whites. Thus, there is an urgency to determine factors that can be used as targets as indicators of obesity. In this study, we attempt to generate a profile of gut and oral microbial clades predictive of disease status in African American (AA) and European American (EA) children. 16S rDNA sequencing of the gut and saliva microbial profiles were correlated with salivary amylase, socioeconomic factors (e.g., education and family income), and obesity in both ethnic populations. Gut and oral microbial diversity between AA and EA children showed significant differences in alpha-, beta-, and taxa-level diversity. While gut microbial diversity between obese and non-obese was not evident in EA children, the abundance of gut *Klebsiella* and *Magasphaera* was associated with obesity in AA children. In contrast, an abundance of oral *Aggregatibacter* and *Eikenella* in obese EA children was observed. These observations suggest an ethnicity-specific association with gut and oral microbial profiles. Socioeconomic factors influenced microbiota in obesity, which were ethnicity dependent, suggesting that specific approaches to confront obesity are required for both populations.

## Introduction

Childhood obesity is a growing worldwide health problem that disproportionately affects ethnic minorities.^1^ In the United States, nearly 13.7 million children and adolescents are obese.^2^ Childhood obesity can lead to several health problems, including cardiovascular disease, type 2 diabetes, certain types of cancer, and coronavirus disease 2019 (COVID-19).^3,4^ The incidence of childhood obesity in African Americans (AAs) (22%) is higher than that of European Americans (EAs) (14.1%). Although the exact cause of this difference is not well established, physical and dietary habits could contribute to changes in the microbial composition and lead to obesity. Additionally, obesity has high heritability, suggesting that genetic factors, in addition to environmental factors, may be involved.^5^ We ascertained various factors that can impact obesity to define disparate rates in AA compared to EA populations in the United States.

The microbiome is influenced by numerous factors, including the environment, geographic location, genetics, and diet. A Western diet, comprised of low fiber, high sugar, and animal fat, influences the intestinal microbiome and has been linked to obesity.^6,7^ The salivary amylase gene (*AMY1*) helps in the digestion of starch by hydrolyzing the ∝-1,4 glycosidic bonds.^8^ AMY1 accounts for 40% to 50% of total protein in human saliva^9,10^ The salivary amylase protein level is correlated with the copy number.^11^ According to one study, variation in copy numbers (CNV) of *AMY1* is directly dependent on the consumption of starch in a diet.^8^ *AMY1* CNV is inversely associated with body mass index (BMI) and obesity.^12^ A recent study showed that *AMY1* CNV is correlated with the composition and function of oral and gut microbiome.^13^

In another recent study, evaluation of gut microbiota differences across ethnicities of US populations revealed that a strong association between ethnicity and abundance of certain taxa was shown where *Christensenellaceae* was associated with BMI.^14^ High BMI has been associated with a decrease in gut microbial diversity.^15^ The obesity-associated gut microbiome can lead to altered colonic gene expression, suggesting an interaction between gene-environmental factors and obesity, as well as other diseases.^16^

There is a paucity of information to understand the factors influencing ethnic disparity in childhood obesity. In the present study, we compared socioeconomic factors such as maternal education, family income, and *AMY1* CNV association with salivary and fecal microbiota in non-obese and obese AA and EA children.

## Results

We evaluated differences in gut and oral microbiomes of 60 children with AA (n = 30) and EA (n = 30) ethnicity. Equal numbers of male and female children were included in both ethnic groups. Participants included non-obese and obese children with median mean age of 8.6 years. There were no significant differences in overall weight, height, BMI *z*-score, and *AMYI* CNV between the 2 groups. However, family income and parental education were significantly different (*P* < .001) between AA and EA populations. The characteristics of the study population are shown in Table 1.

**Table 1.** Characteristics of the study population

**Characteristic**^**a**^	**AA (n = 30)**	**EA (n = 30)**	**Total (N = 60)**	***P*** **value**
**Sex (No.)** Female (%) Male (%)	18 (60) 12 (40)	17 (56.7) 13 (43.3)	35 (58.3) 25 (41.7)	1.00
**Age (y)** Mean (SD)	8.56 (1.57)	8.56 (1.27)	8.56 (1.41)	.83
**Body weight (lb)** Mean (SD)	81 (32.3)	73.3 (17.2)	77.2 (26)	.25
**Height (cm)** Mean (SD)	135 (14)	133 (9.37)	134 (11.8)	.61
**BMI** Mean (SD)	19.6 (4.19)	18.6 (3.05)	19.1 (3.67)	.29
**Non-obese** 5th – 95th percentile^b^ (%) Mean BMI (SD) **Obese** ≥95th percentile Mean BMI (SD)	21 (70) 17.47 (1.81) 9 (30) 24.50 (4.07)	22 (73.3) 17.27 (2.27) 8 (26.7) 22.16 (1.73)	43 (71.7) 17.37 (2.04) 17 (28.3) 23.40 (3.32)	.75 .15
**BMI** ***z*****-score** Mean (SD)	1.237 (1.11)	0.992 (1.35)	1.11 (1.23)	.45
***AMY1* CNV** Mean (SD)	6.89 (2.58)	7.33 (2.36)	7.11 (2.46)	.49
**Annual income ($USD)** < 25,000 (%) 25,001–50,000 (%) 50,001–75,000 (%) >75,001 (%)	23 (76.7) 0 (0) 2 (6.67) 5 (16.7)	2 (6.67) 7 (23.3) 8 (26.7) 13 (43.3)	25 (41.7) 7 (11.7) 10 (16.7) 18 (30)	<.001
**Maternal education** No higher education (%) Higher education (%)	13 (43.3) 17 (56.7)	6 (20) 24 (80)	19 (31.7) 41 (68.3)	<.001

Abbreviations: AA, African American; BMI, body mass index; CNV, variation in copy number; EA, European American.

^a^Data expressed as mean (SD) and *P* < .05 is considered as statistically significant.

^b^No participants were recruited with less than 5th percentile BMI.

## Gut Microbial Diversity Associated with Ethnicity

**Figure 1.**
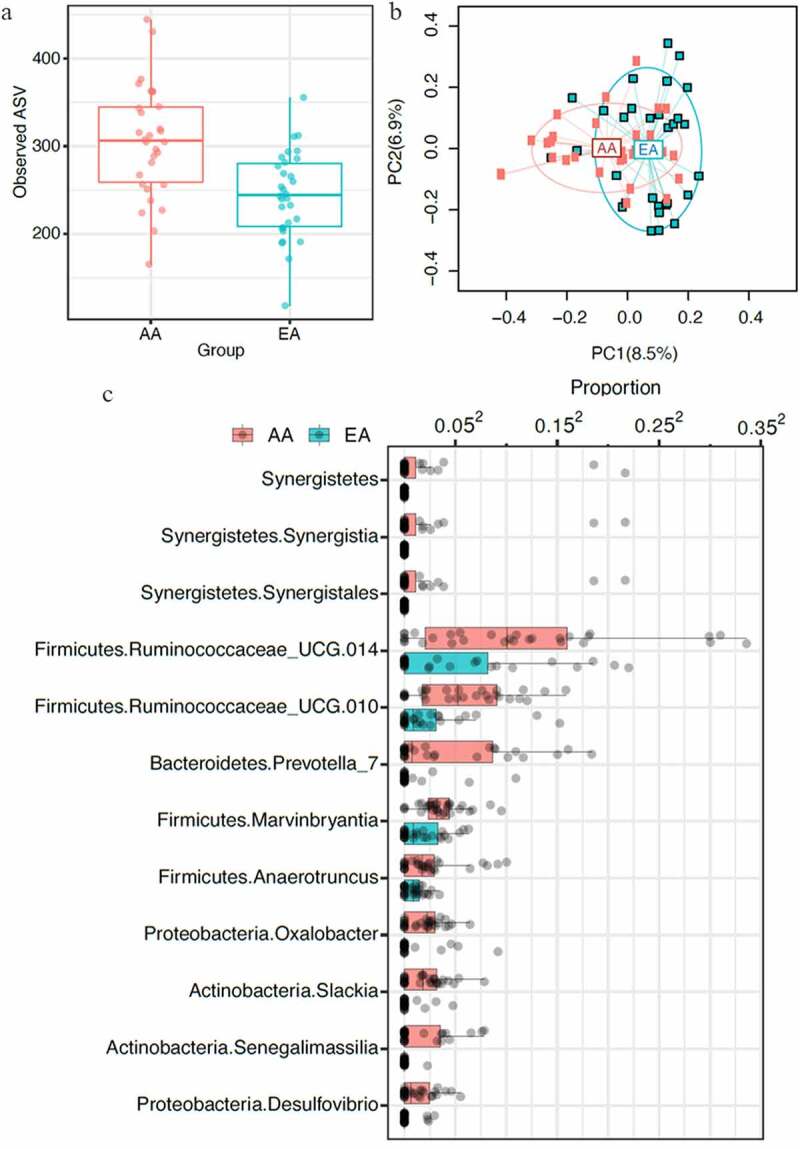
Gut microbiota variability, defined by 16S rDNA sequencing, between children of African American (AA) and European American (EA) ethnic populations (n = 60) showed significant differences between the 2 populations. **A,** Comparison of species richness, alpha diversity, defined by observed amplicon sequence variants (ASV) of AA and EA populations showed significant differences (*p* ≤ .05). **B,** Principal coordinate analysis (PCoA) plot based on the bray-curtis distance matrix constructed using ASVs. The percentage of variability explained by the corresponding coordinate is indicated on the axes. Each point represents a sample – red symbols indicate aa population and blue symbols indicate ea population. The lines indicate vectors representing the relationships between ASVs and each sample category. The ellipses serve a visual guide to group differences. Comparison of beta diversity between aa and ea populations showed significant differences in community structure (*p* ≤ .05). **C,** Differential abundance of taxa in AA and EA populations at 10% false discovery rate. Each dot represents a participant. The relative abundances were plotted on the square-root scale to better visualize the low abundance taxa

As ethnicity plays a vital role in microbial differences in the human microbiome, we first evaluated differences between the microbiome of AA and EA children. Also, a comparison of oral and gut microbiota showed differential structure and composition, with most taxa being differentially expressed in both sites (Figure S1). We compared gut microbial diversity between AA and EA participants. There were significant differences in the alpha and beta diversity between AA and EA participants (Figure 1a and b), supporting the previously published observations that microbial differences were dependent on race.^7,17,18^ Differential abundance analysis showed a higher abundance of *Ruminococcaceae* in AA children compared to EA children (Figure 1c). Race-associated bacterial differences were observed in many genera, including *Anaerotruncus, Desulfovibrio, Marvinbryantia, Oxalobacter, Prevotella, Senegalimassilia*, and *Slackia*, all showing higher abundance in AA children than EA children (Figure 1c, at FDR [false discovery rate] <0.1).

## Oral Microbiota Differs By Ethnicity

**Figure 2.**
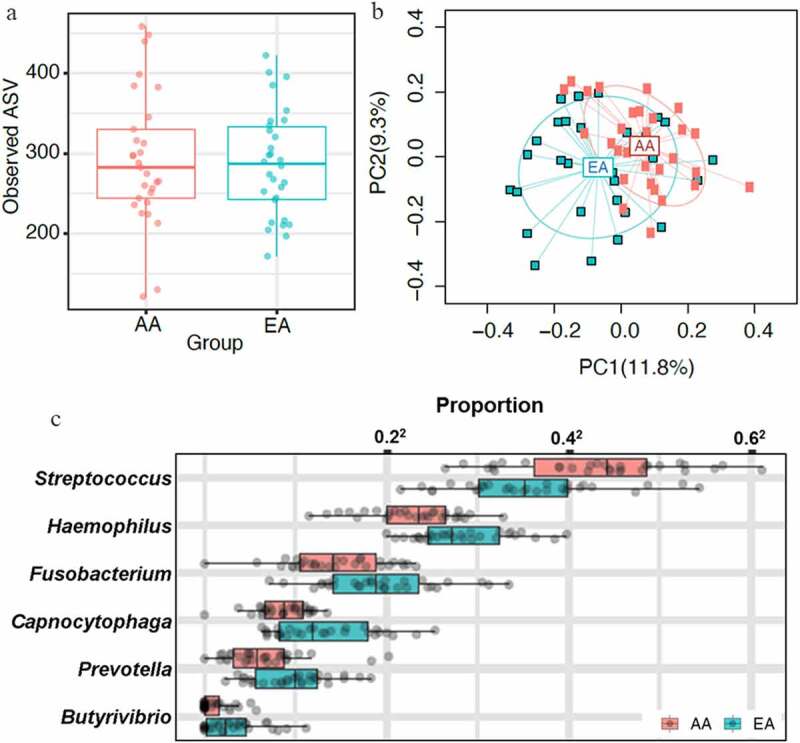
Oral microbiota comparison between AA and EA populations (n = 60) showed similar alpha diversity with considerable difference in beta diversity with ethnicity-specific taxa. Oral microbiota was sequenced using saliva samples. **A,** Alpha diversity was analyzed by observed ASVs. No significant difference in alpha diversity of oral microbiota was observed between AA and EA groups. **B,**Bray-curtis distance matrix for beta diversity between AA and EA populations was analyzed using permanova. Comparison between AA and EA groups showed a significant difference in beta diversity (*p* ≤ .05). **C,** Genus-level differentially abundant taxa in AA and EA groups at 10% false discovery rate were presented. The relative abundances were plotted on the square-root scale to better visualize the low abundance taxa. *Streptococcus* was present with an increased abundance in AA children compared to EA children, and 5 genera, *butyrivibrio, capnocytophaga, fusobacterium, haemophilus*, and *prevotella*, were abundant in EA but no in AA groups

Next, we determined whether oral microbiota has any ethnic specificity. The alpha diversity was similar between the AA and EA groups (Figure 2a and b). But comparison of oral bacterial diversity and composition showed a considerable difference in beta diversity between AA and EA ethnicity (Figure 2b). Differential abundance analysis showed an increased abundance of genera belonging to *Firmicutes* and *Actinobacteria* in the AA group and a higher abundance of *Proteobacteria, Fusobacteria*, and *Tenericutes* in the EA group (Figure S2). *Streptococcus* was present with an increased abundance in AA children compared to EA children, whereas *Butyrivibrio, Capnocytophaga, Fusobacterium, Haemophilus*, and *Prevotella* were reduced in AA compared to EA children (Figure 2c). *Prevotella* is a key bacterium that showed high abundance in the stool samples of AA children, but were at low levels in the oral microbiome of EA children.

## Gut Microbiota is Associated With Obesity in AA Population

**Figure 3.**
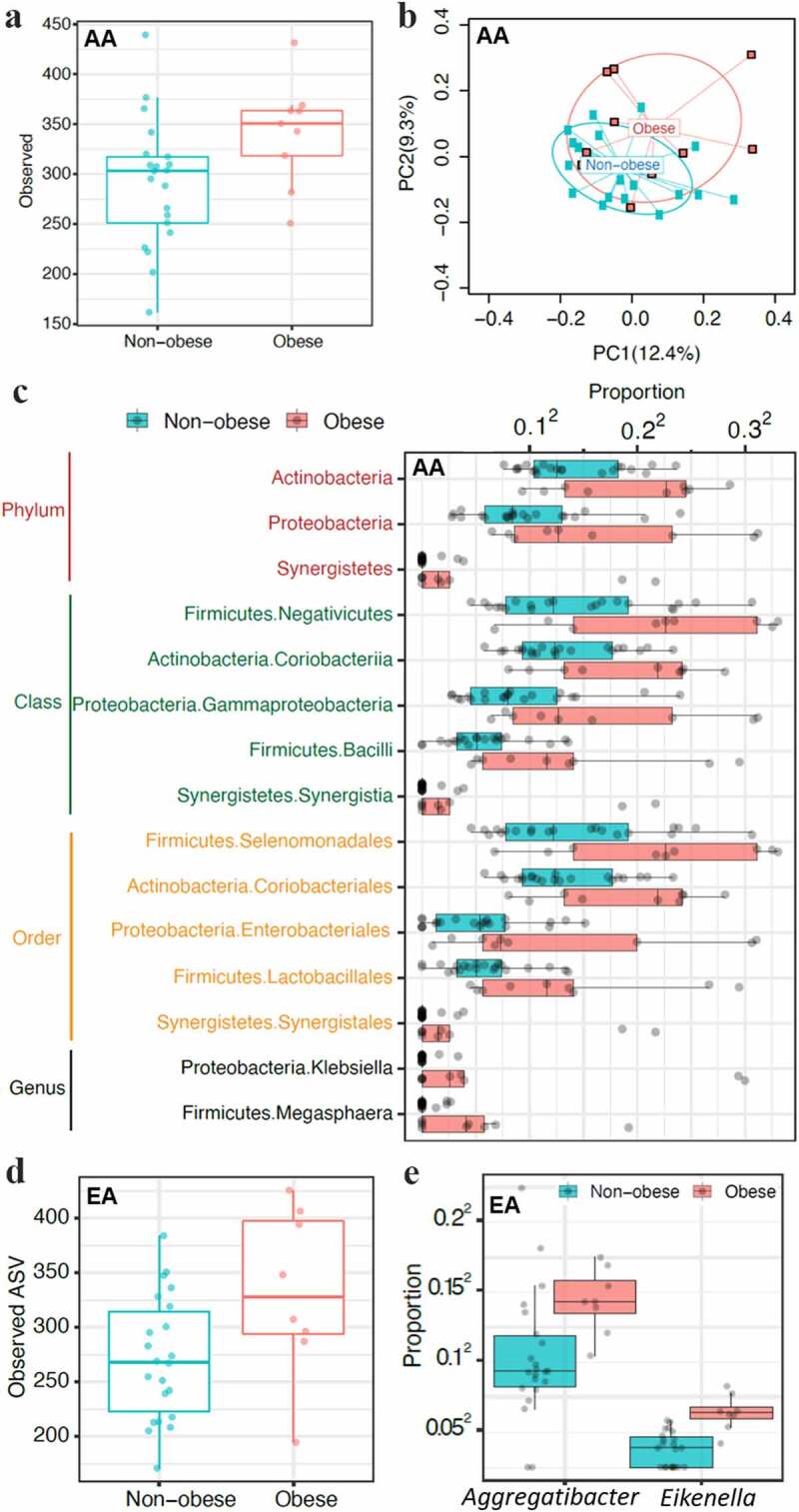
Microbiota differences between obese and non-obese children showed ethnicity-dependent associations. Oral microbial diversity was associated with obesity in EA children and gut microbial diversity in aa children. **A-C,** Gut microbiota differences in stool samples between obese and non-obese AA children. **A,** Alpha diversity measured by observed ASVs showed increased diversity in obese children compared to non-obese children. **B,** Beta diversity (bray-curtis distance) showed significant differences in gut microbiota between obese and non-obese AA children (*p* ≤ .05). **C,** Differentially abundant taxa in obese and non-obese AA children (n = 30). Children from the EA group did not show major differences in gut microbiota alpha and beta diversity as well as differences in taxa abundance between obese and non-obese participants (not shown). **D-E,** Oral microbial diversity was associated with obesity in EA children only (n = 30). **D,** Salivary microbial alpha-diversity comparison between obese and non-obese EA children showed obesity was associated with increased diversity. **E,** Genera *Aggregatibacter* and *Eikenella* abundance was increased in obese compared to non-obese EA children. Children from the AA group did not show significant differences in alpha, beta, and taxa diversity in salivary microbiota (not shown)

The initial groups of normal and overweight participants did not show any substantial differences (Figure S3), so they were divided into only obese and non-obese groups (normal and overweight groups were combined into non-obese). When both groups were analyzed individually, a substantial difference in alpha diversity (number of observed ASVs [amplicon sequence variants]) and beta diversity between obese and non-obese children was observed only in AA children (Figure 3a and b). The major microbiota difference was observed in the presence of rare and less abundant taxa. Differential abundance analysis of the AA group revealed the association of obesity with the abundance of taxa from *Actinobacteria, Proteobacteria*, and *Firmicutes* and notably *Klebsiella* and *Magasphaera* in obese AA children (Figure 3c, at FDR <0.1). Children from the EA group did not show a substantial difference in taxa abundance between obese and non-obese participants, suggesting that obesity-associated gut microbiome may be race dependent.

## Oral Microbiota is Associated With Obesity in EA Children

An analysis on the association of oral microbiota with obesity did not show any considerable difference in alpha and beta diversity, and no taxa were found to be substantially associated in the AA group. On the other hand, a considerable difference in alpha diversity (observed ASV numbers) between obese and non-obese children was observed in the EA group (Figure 3d). Abundance of *Aggregatibacter* and *Eikenella* in the EA group were associated with obesity (Figure 3e, at FDR <0.1). These results suggest that obesity-associated oral microbiota may also be race dependent.

## AMY1 Copy Numbers Not Correlated With Obesity

**Figure 4.**
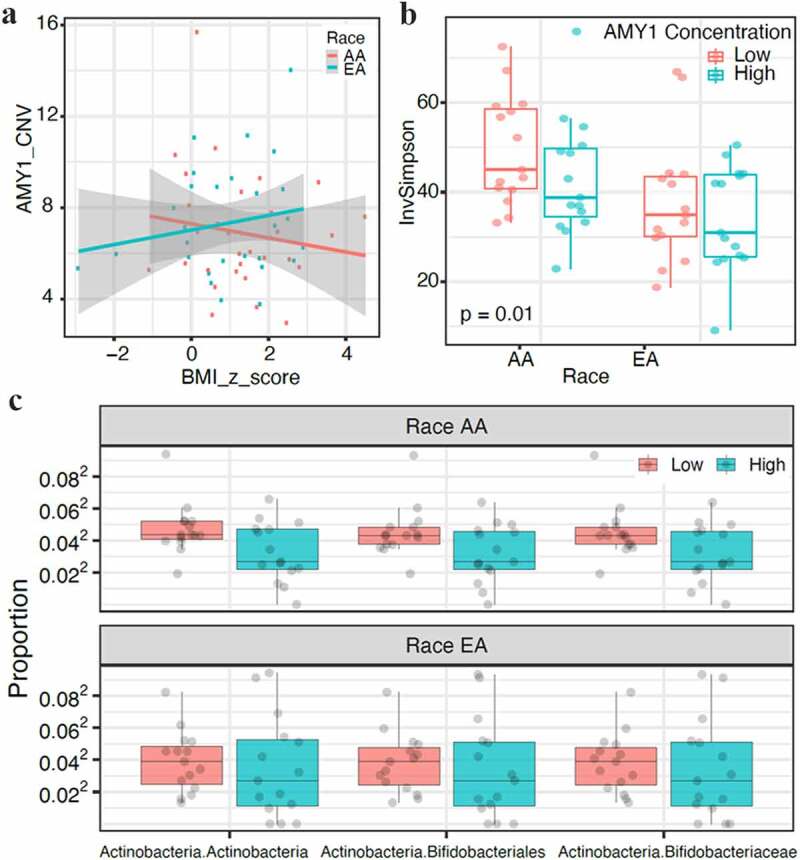
Correlation of *AMY1* copy numbers (CNVs) with body mass index (BMI) and gut microbiome. *AMY1* is not associated with BMI. **A,** AMY1 and BMI *z*-score did not show any correlation in EA and AA populations (n = 60). **B,** Alpha diversity measured by inverse simpson index (inv simpson) showed a significant difference in gut microbial diversity between low and high levels of *AMY1* in AA children (*p* = .01) but not EA children. **C**, *AMY1* levels reflected taxa diversity in the gut microbiome with low abundance of *Bifidobacteriaceae* representing *Actinobacteria* in *AMY1*-high children when compared to amy1-low children in both populations (*p* ≤ .05)

Recently, some studies have shown an association between salivary amylase enzyme and obesity, while others did not confirm this association.^11,12,19,20^ In this data set, there was no significant correlation between BMI *z*-score and *AMY1* CNV in either population (Figure 4a). Also, no significant association was observed between oral microbiota and *AMY1* CNV in either population. However, an increase in alpha diversity of gut microbiota was observed in children with low *AMY1* CNV compared to high CNV (Figure 4b). Higher abundance of *Bifidobacteriaceae* representing *Actinobacteria* was present in children with low *AMY1* CNV compared to high *AMY1* in both populations (Figure 4c). These data suggest an association of gut microbiota with *AMY1* even though there was no correlation between the BMI *z*-score and *AMY1* CNV in the present data.

## Role of Socioeconomic Factors in Obesity and Correlation to Microbiota

**Figure 5.**
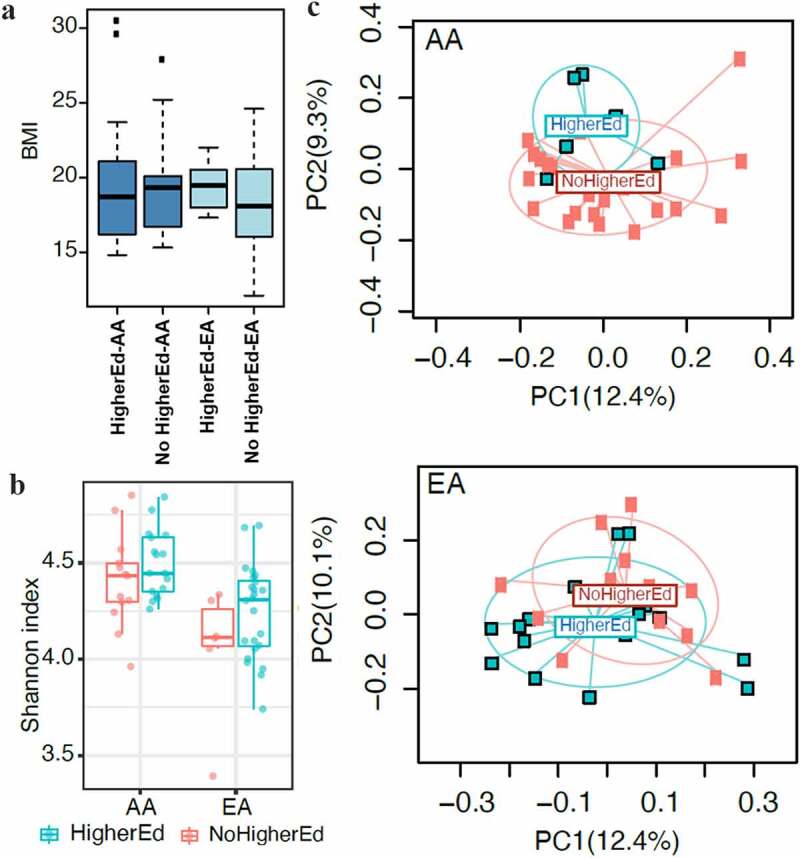
Educational status of parents did not impact BMI but did influence gut microbial diversity. Families with 1 parent with college degree were categorized as higher education. **A**, Correlation between educational status of parents and BMI *z*-score of children was not significant in either populations. **B**, Education levels of parents correlated with a nonsignificant but an increased trend of gut microbiota alpha diversity. **C**, Beta diversity in children of both groups showed significant differences associated with their parent’s educational status (*P* = .02). However, no abundance of specific taxa in gut microbiota was observed based on the educational status of the parents in both populations. No significant associations between educational status and oral microbiota were observed between populations (not shown)

A report by The Centers for Disease Control and Prevention (CDC) in 2017 suggested that the prevalence of obesity can differ based on race, income, and education.^21^ Generally, lower-income groups and non-college graduates have a higher prevalence of obesity.^21^ Here, we tested whether obesity in the children of AA and EA groups is dependent on the income and education status of parents. Families with at least 1 parent with a college degree or any degree after high school were classified as having higher education. There was no significant difference in the BMI *z*-score in the 2 populations based on the education of parents (Figure 5a). As shown in Table 1, 77% of the AA group and 7% of the EA group had an annual household income of less than $25,000, showing a considerable difference in overall income between the 2 populations. Using a cutoff of $50,000 as low annual income allowed us to classifying 30% of children in the EA group as low income. Both AA and EA groups did not show a significant association of higher BMI *z*-score with an annual household income of more than $50,000 (Figure S4).

A comparison of gut microbial composition based on the education of parents showed that a higher education status of parents was associated with a nonsignificant trend toward an increase in microbial diversity as measured by the Shannon index (*P* = .06) (Figure 5b). Interestingly, higher education was more strongly associated with beta diversity in the AA group than the EA group (Figure 5c). However, no abundance of specific taxa was observed based on the educational status of parents. Also, no associations between the education status and the oral microbiota were found.

**Figure 6.**
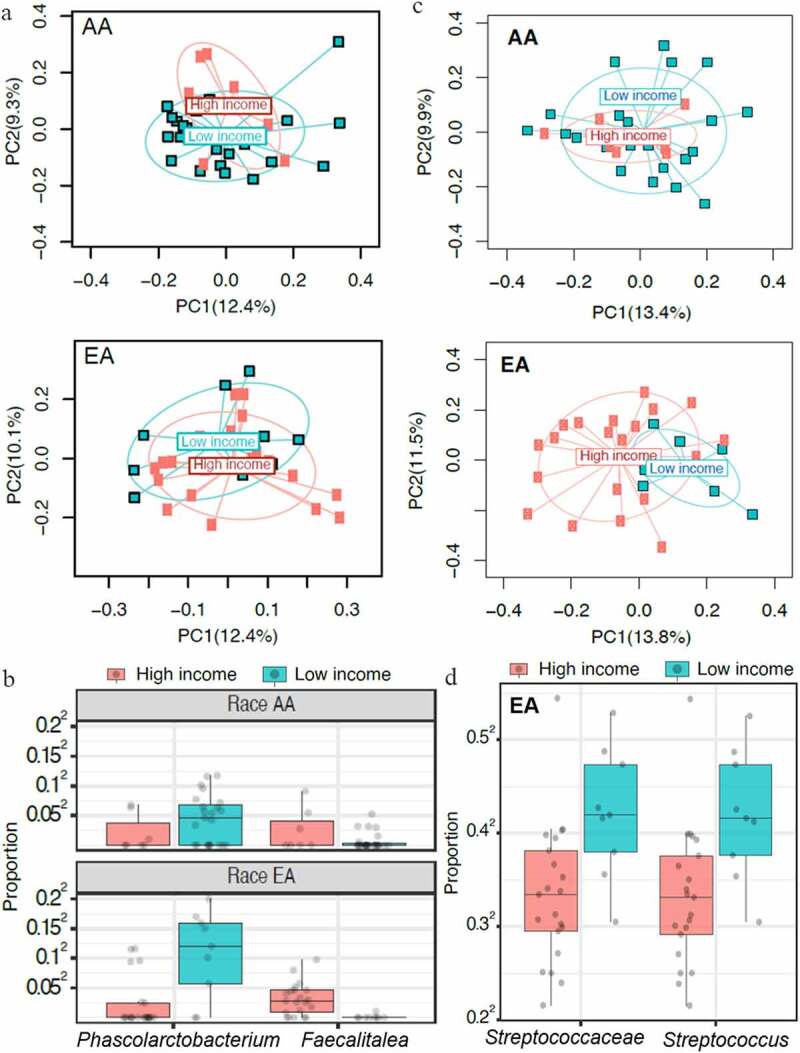
Family income strongly influences gut and oral microbiota based on ethnicity. A cutoff of $50,000 per family was used as low annual income. **A**, In both AA and EA children belonging to higher income families, an increased beta diversity in gut microbiota was shown (*P* = .03). No correlation between income and gut microbial alpha diversity was observed in both groups. **B**, Both groups had increased abundance of gut *Phascolarcobacteria*, with a decrease in *Faecalitalea* (both belonging to the phylum Firmicutes) in families with low income (*P* ≤ .05). Oral microbial diversity differed based on the income of the EA and AA families. **C**, Beta diversity as determined by Bray-Curtis distance was associated with income in both groups (*P* = .001). **D**, Differentially abundant taxa analysis of oral microbiota showed increased abundance of *Streptococcus* in EA children from low-income families. No differentially abundant taxa were associated with income in AA children (not shown)

Next, we determined whether income had any correlation with gut or oral microbiota in both populations. While there was no significant difference in the gut microbiota’s alpha diversity, the beta diversity was significantly associated with income (Figure 6a). Children from low-income families from both AA and EA populations had lower levels of *Faecalitalea* and higher *Phascolarcobacterium*, both belonging to the *Firmicutes* phylum (Figure 6b, at FDR <0.1). For the oral microbiota, no significant association was observed in the alpha diversity, though the microbial community structure was significantly different, as observed by a difference in beta diversity (Figure 6c). Interestingly, a strong association was evident only in the EA group. Differential abundance analysis showed an association of a higher abundance of *Streptococcus* in the EA group with low income only (Figure 6d). These data suggest that a microbial association between obesity and socioeconomic factors is dependent on ethnicity.

## Discussion

he recent COVID-19 pandemic has brought into focus the disproportionate burden of illness, with higher mortality rates in minority populations than the White population, according to the CDC.^22^ Even though the cause of this major disparity remains unclear, comorbidities have been suggested as the primary reason. An association between gut microbiota and obesity has been documented.^16,23,24^ There is an evidence of increased obesity prevalence in minorities, which starts during childhood. Hence, an insight into the factors that strongly influence oral and gut microbiome is essential to understand the cause of the variation in obesity.

There is limited information available on the gut and oral microbial profile in obese children of AA and EA ethnicity, and reports have not been consistent.^25,26^ One study reported an increase in *Enterobacteriaceae* in obese children compared to children of healthy weight, while another reported an increase in *Bacteroides* in obese Mexican children. In a study of a small adult AA population, a variability of gut microbiota was observed according to ethnicities with heritable taxa.^14^ A recent study suggested that heritability of certain microbes from an overweight mother to infant predisposes to obesity in adulthood.^27^ *Magasphaera*, a vaginal microbe, has been associated with obesity in adults.^23^ We observed an increased abundance of *Magasphaera* in obese children of AA descent. Similarly, *Coriobacteriaceae* and *Runmicoccaceae* observed in infants born to overweight mothers were increased in obese AA children.^27^ Whether the presence of these microbes in obese children is hereditary or due to mode of delivery is unclear, as birth mode was not collected in this study. The differences in BMI-associated taxa in children of AA and EA descent could also be due to the fact that heritable taxons of bacteria vary by ethnicity.^14^ Indeed, children of EA and AA descent showed variability in gut microbiome, as observed by alpha and beta diversity. AA children had a higher abundance of *Anaerotruncus, Desulfovibrio, Marvinbryantia, Prevotella, Slackia, Senegalimassilia, Runmicoccaceae*, and *Oxalobacter*, and the final 2 have been associated with obesity.^27,28^ *Slackia* spp are gut-associated bacteria that have been suggested to play a role in host lipid xenobiotic metabolism,^29^ and its abundance may be dependent on diet. Sulfate-reducing bacteria, *Desulfovibrio*, are reported to be associated with a high-fat diet,^30^ and its co-occurrence in the gut with *Prevotella* has been speculated to work synergistically for degrading mucin.^31^ *Desulfovibrio* has been reported in autistic children.^32^ A study on colorectal cancer showed that the richness of *Desulfovibrio* in the gut was associated with AA patients.^33^ In the present study, *Desulfovibrio* did not markedly correlate with obesity. Also, the abundance of *Anaerotruncus, Marvinbryantia*, and *Senegalimassilia*, not known to be related to obesity, in AA children was a novel observation. However, no major difference in the taxa between obese and non-obese EA children was somewhat surprising. Reasons for this could include mode of birth and diet. A previous study in AA and EA populations did show an increase in *Prevotella* in AAs, although that study was dependent on diet.^7^ An increase in *Prevotella* has been correlated with enhanced glucose metabolism in the gut.^34^ A recently published study showed that the association between gut *Prevotella* and BMI was most noticeable among AA adults.^35^ However in the present study, *Prevotella* in the gut of AA children did not associate with BMI, suggesting that the *Prevotella*-BMI association may be dependent on age.

Interestingly, the oral microbiome of EA children showed microbial variability between obese and non-obese children, whereas AA children did not show that variability. Salivary microbiota in obese EA children showed an abundance of *Aggregatibacter* and *Eikenella*, both associated with periodontitis and obesity.^36–38^ *Aggregatibacter* has been described as an oral pathogen associated with obesity in AA and Japanese adults.^37^ *Eikenella* was found to be increased in obese Brazilian patients with chronic periodontitis.^38^ Hence, the changes in oral microbiome may also have regional specificity. Gupta et al^39^ showed how geography, ethnicity, and subsistence play a role in the diversity of human microbiome composition. While many opportunistic pathogenic species, such as *Streptococcus, Butyrivibrio, Capnocytophaga, Fusobacterium, Haemophilus*, and *Prevotella*,^40,41^ showed differences in salivary microbiome between AA and EA children, *Streptococcus* are the only bacteria that differentiated with higher abundance in AA children, supporting a previous study of its increased presence in low-income AA populations.^38^ The present data suggest that the oral microbiome in obesity is much more relevant for EA children than AA children.

Reduced *AMY1* copy numbers have been associated with increased BMI and obesity risk in European and Asian populations.^11,42^ In this study, no association between *AMY1* CNV and BMI was observed in either population. The salivary microbiome in obese children has shown low diversity similar to obese children with type 2 diabetes.^43^ We did not observe any association between *AMY1* CNV and oral microbiome in either population. However, there was an inverse relation between copy numbers and gut microbial diversity. Low *AMY1* CNV was associated with increased gut microbial diversity in the AA population and obese AA children, supporting previous observations of low CNV association with gut diversity. Low *AMY1* CNV has been associated with obesity and an increased abundance of *Prevotella* in Mexican children.^44^ In this study, a higher abundance of *Bifidobacteriaceae* was associated with low *AMY1* CNV, suggesting that these associations could be ethnic-specific. Even though no association between *AMY1* and oral microbiome was observed, salivary microbiota differed between the 2 populations and was markedly different between obese and non-obese children.

We then determined whether microbial variability can be explained by differences in socioeconomic factors within the 2 studied populations. A racial disparity in obesity has been reported, with a higher incidence of obesity in Blacks than Whites among individuals with higher education levels.^45^ In this study, families with parents having a college degree showed an increase in beta diversity in AA children. It is assumed that education would help parents pick healthier dietary options. However, since education levels were not associated with BMI, it suggests that increased awareness about obesity and diet is required.

Poverty has been associated with an increased burden of obesity.^46^ In this study, AA families with a higher annual income showed an association with obesity in children. However, 76% of the AA families had low income; thus, more families with higher income need to be studied. On the other hand, income had no correlation with obesity in the EA group, though increased beta diversity was associated with families with a higher annual income. Low income was associated with increased abundance of *Phascolarcobacterium*, a microbe present in the human gastrointestinal tract.^47^ This bacterium uses succinate produced by other bacteria and leads to production of propionate; thus, it is a microbe of health. However, when increased in abundance due to a high-fat diet, it has been associated with metabolic disorders in rats.^48^ On the other hand, *Faecalitalea*, a butyrate-producing *Firmicute* and reported to be 1 of the baseline species in healthy human gut,^49^ was decreased in children from low-income families. This suggests that income-based choice of diet may have a substantial influence on gut microbiota. However, the study is limited by low numbers of children in each category as well as differences in income and education levels between the 2 studied ethnic groups, impacting the ability to detect associations. Further limitations include a lack of other data, including metagenomics, transcriptomics, and metabolomics, that can provide an in-depth insight into ethnic-specific factors associated with obesity. Further studies are required to confirm these associations in ethnic populations.

## Conclusions

Observations from this study suggest that, while children of AA descent show an association with gut microbiota and obesity, children of EA descent show an association between salivary microbiome and obesity. This means that in order to more accurately show that gut microbes are associated with obesity, different ethnic groups need to be studied, and any solution should be adapted to different ethnic populations. The study has limitations in the numbers of participants in various associations tested. Thus, further studies with increased numbers of children from the 2 populations are required to determine specific associations to provide ethnic-specific solutions to the obesity.

## Materials and Methods

### Participants and Anthropometric Parameters

All patients fulfilled the established exclusion and inclusion criteria. The study was approved by the institutional review board of Auburn University. Written informed consent was received from all participants and their parents prior to inclusion in the study. The inclusion criteria to admit participants in the study were age between 6 and 10 years and either EA or AA ethnicity. The exclusion criteria included children with major health problems associated with obesity, including cardiovascular disease or type 2 diabetes, and children who were taking antibiotics. The AA children (n = 30; 18 female, 12 male) and EA children (n = 30; 17 female, 13 male) residing in the state of Alabama, USA, were enrolled with the consent of their parents. Children with the major health problems outlined above were excluded from the study. The characteristics of the study population are given in Table 1.

Participants’ height was measured to the nearest 1/8 inch and weight to the nearest 4 ounces,^26^ and measurements were used to calculate BMI *z*-score,^12^ to assess for obesity, and to approximate the ideal weight for a person, considering both sex and age.^50^ The children were classified as normal weight, overweight, and obese based on CDC guidelines (https://www.cdc.gov/obesity/data/childhood.html). Based on BMI cutoff, non-obese groups are from the 5th to 95th percentile derived by combining the normal weight (5th to <85th percentile) and overweight (85th to <95th percentile) categories. Participants with a BMI ≥95th percentile were considered obese.

### Sample Collection

Saliva collection was done using a saliva collection kit (DNA Genotek Inc., Ontario, Canada). Before the collection of saliva, children were asked to briefly rinse their mouth to avoid the influence of any food particles present. Fecal samples were collected using a stool collection kit (LPCO diagnostics, MS). Both saliva and fecal samples were transported immediately to the laboratory (using a temperature-controlled container) and stored at – 80°C.

### AMY1 Gene Copy Number

DNA was extracted from saliva using the PrepIT.L2P method (DNA GenoTek, Ontario, Canada). Copy numbers for the *AMY1* gene were done by polymerase chain reaction (PCR) (eMethods).^12^ Briefly, using a digital PCR (QuantStudio 3D Digital PCR) comprising 2 TaqMan assays (Hs07226361_cn FAM-labeled and Hs07226361_cn, FAM-labeled). For the PCR reaction, 14.5 μL of reaction mixture containing 7.25 μL of PCR Master Mix, 6 μL of diluted DNA (10 ng/μL), 0.725 μL of 20× RNase P, and 0.725 μL of 20× *AMY1* were loaded into the PCR chip. PCR was performed by setting the appropriate parameters (initial denaturation at 96°C for 10 min, 39 cycles of 60°C for 2 min, and 98°C for 30 sec, followed by 1 cycle of 60°C incubation for 2 min, and then 4°C hold). After PCR reaction, the PCR chip was subjected to QuantStudio 3D Digital scanning and analysis (QuantStudio 3D Analysis Suite Software).

### 16S rDNA Sequencing

Fecal and oral samples were subjected to genomic DNA isolation using the MoBio PowerSoil Kit (QIAGEN, USA). Genomic DNA was subjected to PCR amplification of the V3-V5 region of 16S rDNA using 50 ng cDNA and 0.3 µM barcoded primers (V3_F: TCGTCGGCAGCGTCAGATGTGTATAAGAGACAGCCTACGGGAGGCAGCAG; V5_R: GTCTCGTGGGCTCGGAGATGTGTATAAGAGACAGCCGTCAATTCMTTTRAGT) with Kapa HiFi Hotstart Ready Mix (Kapa Biosystems). Samples were pooled to equal concentrations, then sequenced for 16S rRNA using the MiSeq 600 cycle v3kit (Illumina Inc.). Sequence files were denoised by DADA2 into ASVs;^51^ the SILVA database^52^ was used to assign taxonomy to ASVs using Naïve Bayes classifier, and FastTree^53^ was used to construct the phylogenetic tree among ASVs. Following quality control, we obtained 7,243,786 high-quality reads and a total of 12,842 ASVs. The median (range) of the sequencing depths for fecal and oral samples were 56,439 (9,099–118,425) and 56,559 (3,033–133,629), respectively.

### Statistical Analysis

#### Anthropometric Data Analysis

Growth in children occurs until approximately 20 years of age. Therefore, the BMI *z*-scores were calculated using SPSS macro-based World Health Organization growth reference 2007 data adjusted for age and sex.^54^

#### AMY1 CNV

Calculation of *AMY1* gene CNV was analyzed by the ratio between *AMY1* and the RNAase P gene CNV obtained from 3D digital PCR analysis in an excel spreadsheet. The anthropometric data were compared between mean values of EA and AA groups by independent sample *t* test using SPSS (version 24, IBM, Armonk, NY, USA). The results in Table 1 are expressed as mean (SD), and *P* < .05 was considered statistically significant.

#### Microbiome Analysis

The microbiome data analysis was conducted for alpha diversity, beta diversity, and taxa abundances. We have tested the association between race, BMI, *AMY1* CNV, parental education and parental income (referred as “variables of interest”), and oral and stool microbiomes. To improve statistical power, we first pooled both EA and AA participants in association tests adjusting for race. Next, we conducted association tests in both the EA group and the AA group to identify potential race-dependent associations.

#### Alpha Diversity Analysis

Three alpha diversity metrics – observed ASV number,^53^ Shannon index, and Inverse Simpson index – were calculated based on the rarefied ASV counts to control for sequencing depth difference (“estimate richness” function in Bioconductor package phyloSeq).^55^ Observed ASV number is a species richness measure, while the Shannon index and Inverse Simpson index measure overall diversity, taking into account both species richness and evenness, with the latter putting more emphasis on abundant species. A simple linear model was used to test the association between alpha diversity measures and variables of interest, adjusting for other covariates when necessary.

#### Beta Diversity Analysis

Unweighted and weighted UniFrac and Bray-Curtis (BC) distances were constructed using the ASV table and the phylogenetic tree (R package, GUniFrac).^56^ Rarefaction was performed on the ASV table before calculating distances. Based on these distance matrices, Permutational Multivariate Analysis of Variance (PERMANOVA) was used to test for an association between variables of interest and the overall microbiota composition, adjusting for other covariates when necessary (R package, vegan).^57^

#### Differential Abundance Analysis

Only taxa with a presence in more than 10% of the samples and with a relative abundance greater than 0.2% in at least 1 sample were tested. This reduced the total number of tests. The count data was normalized by the geometric mean of pairwise ratios size factor.^58^ To identify differentially abundant taxa, permutation tests (999 permutations) were performed for each taxon, using the F-statistic of a linear model (square-root transformed, normalized abundance as the outcome) as the test statistic.^59^ FDR control (B-H procedure) was used to correct for multiple testing at each taxonomic level, and FDR-adjusted *P* values or *Q* values <.10 were considered significant (“p.adjust” in R). For non-multiple hypothesis testing,   *P*<.05 was considered statistically significant.

## Supplementary Material

Supplemental Material
