# Supplementary material for: Ethnic variability associating gut and oral microbiome with obesity in children
Source: Gut Microbes. 2021 Feb 17;13(1):1882926. doi: 10.1080/19490976.2021.1882926 (PMC7894456; doi:10.1080/19490976.2021.1882926)

**Supplementary Materials**

**Figure S1.** **The fecal and saliva microbiome have very different structures and compositions.** Principal coordinate plots based on unweighted and weighted UniFrac distances. PERMANOVA analysis shows the significant difference between fecal and saliva microbiome on unweighted UniFrac and weighted UniFrac (A), P<0.05. The percentage of variability explained by the corresponding coordinate is indicated on the axis. Each point represents a sample with red and green color indicating the fecal and saliva samples respectively. The lines connecting to the centroid and the ellipses do not represent any statistical significance but rather serve a visual guide to group differences. B) Genus level differential abundance shows significant differences between fecal and saliva samples (FDR <0.1).


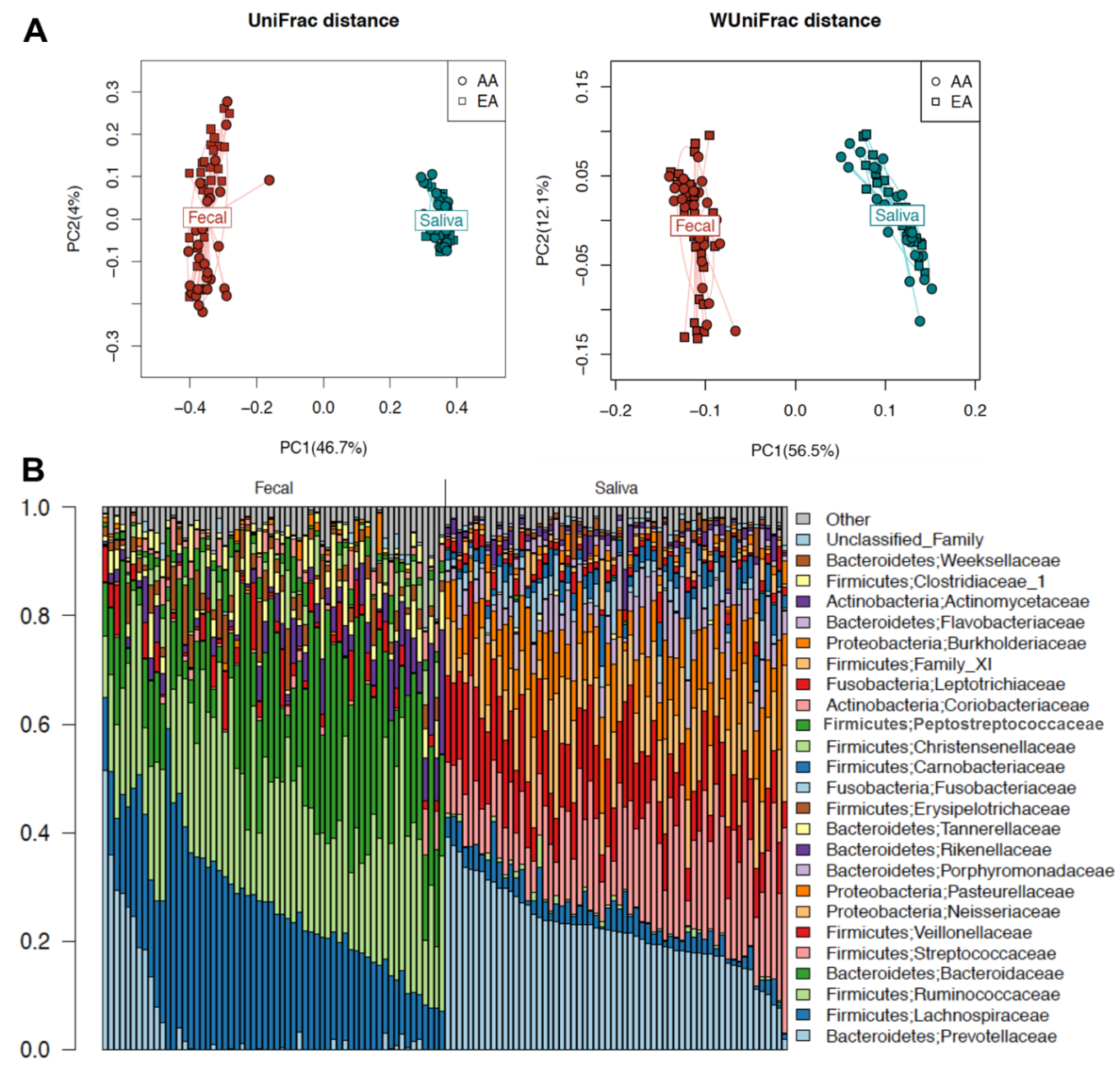


**Figure S2.** **Oral microbiota taxa level comparison between AA and EA groups (N=60).** Phylum, class, order, family and genus level differential abundance of taxa in AA and EA groups.


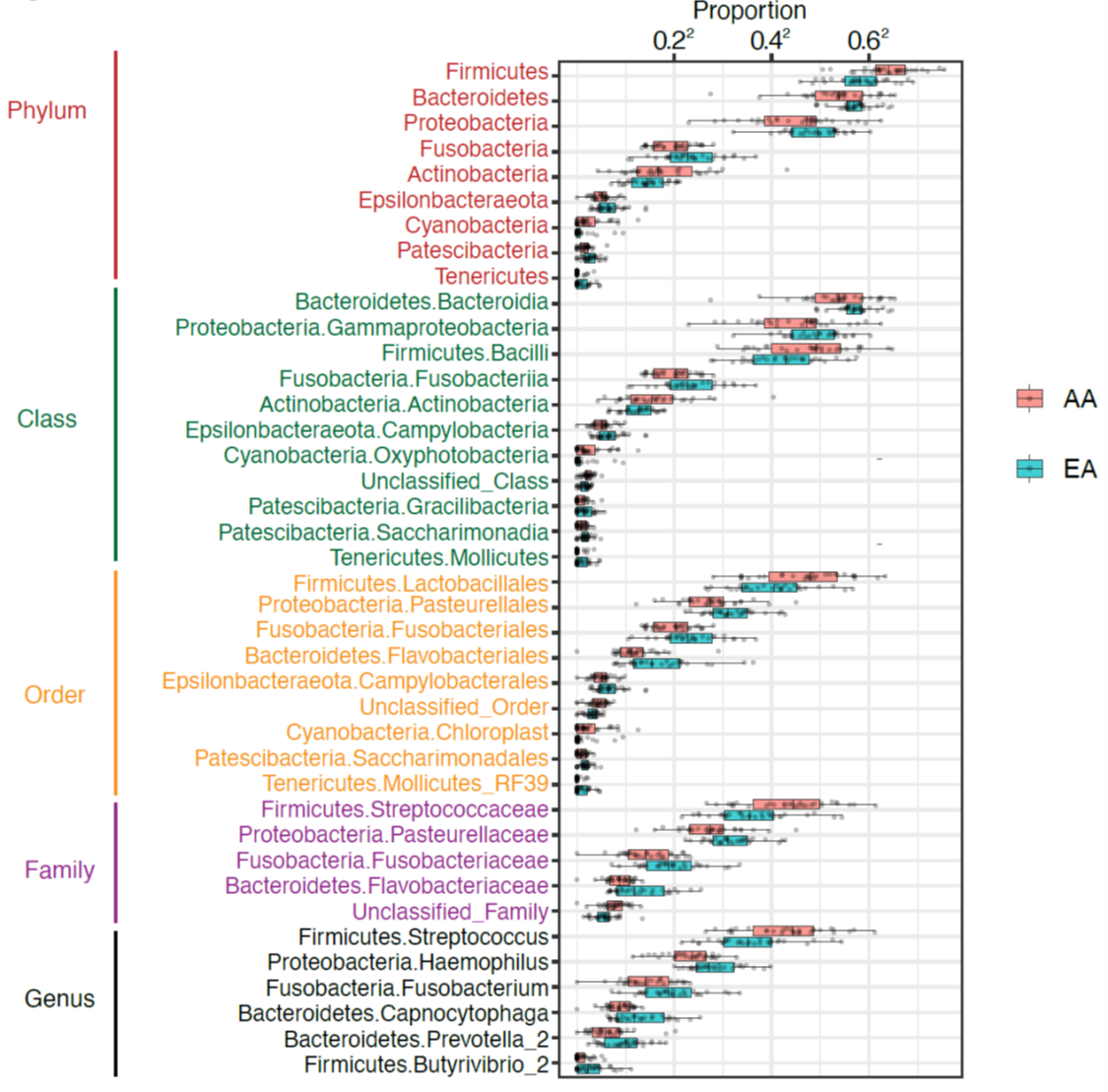


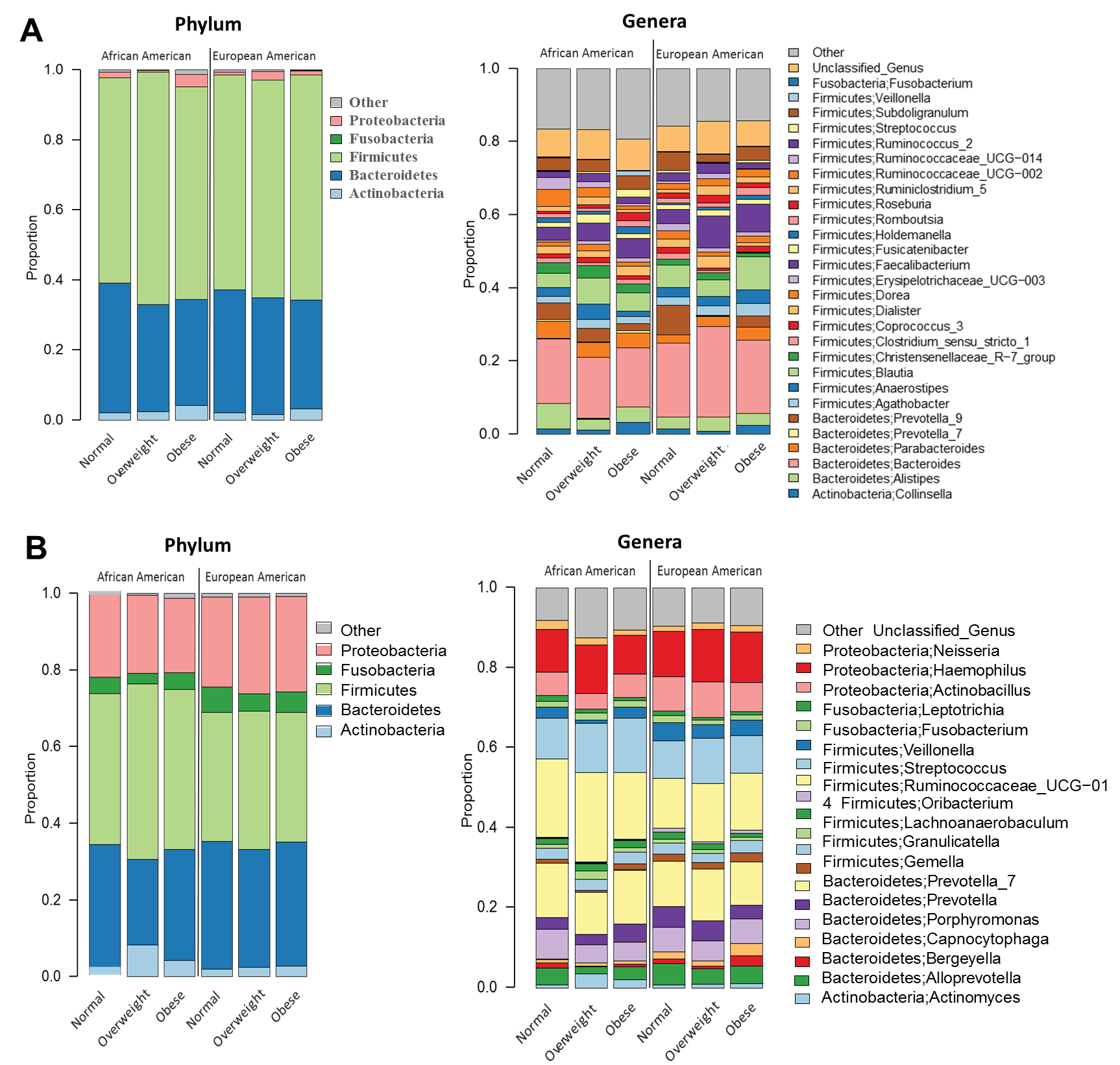
**Figure S3. Microbial diversity did not show any taxonomical level differences when categorized into normal, overweight, and obese using BMI *z*- score.** A) Distribution of various phyla and genera in fecal samples and B) Distribution of various phyla and genera in saliva samples of all children in the study.

**Figure S4. Association of BMI-z score with family income variations.** BMI was associated with high income in families of AA children, P= 0.06.


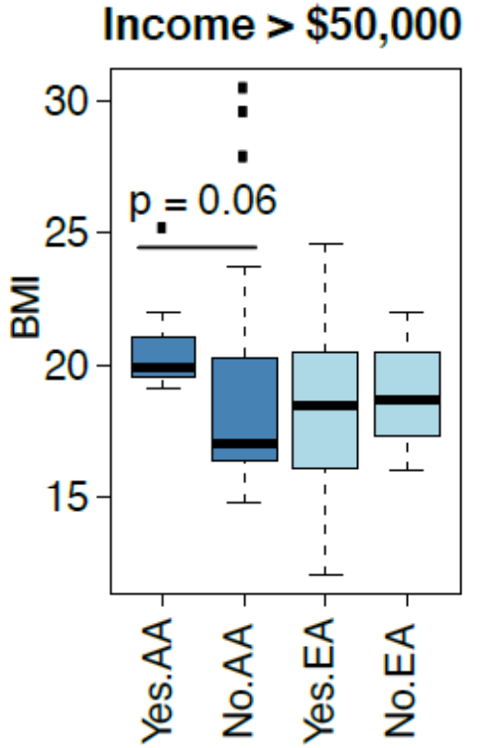

Supplement: Supplemental Material [file KGMI_A_1882926_SM2924.docx]
